# Persistent Autobiographical Amnesia: A Case Report

**DOI:** 10.1155/2007/534043

**Published:** 2007-02-12

**Authors:** C. Repetto, R. Manenti, V. Sansone, M. Cotelli, D. Perani, V. Garibotto, O. Zanetti, G. Meola, C. Miniussi

**Affiliations:** ^1^IRCCS San Giovanni di Dio FBFBresciaItaly; ^2^Vita Salute University and San Raffaele Scientific InstituteMilanItaly; ^3^Department of NeurologyPoliclinico San DonatoUniversity of MilanItaly; ^4^Cognitive ScienceUniversity of TurinItaly; ^5^IRCCS San Raffaele HospitalMilanItaly; ^6^Department of Biological Sciences and BiotechnologiesUniversity of BresciaItaly

**Keywords:** Functional retrograde amnesia, FRA, memory, autobiographic amnesia

## Abstract

We describe a 47-year-old man who referred to the Emergency Department for sudden global amnesia and left mild motor impairment in the setting of increased arterial blood pressure. The acute episode resolved within 24 hours. Despite general recovery and the apparent transitory nature of the event, a persistent selective impairment in recollecting events from some specific topics of his personal life became apparent. Complete neuropsychological tests one week after the acute onset and 2 months later demonstrated a clear retrograde memory deficit contrasting with the preservation of anterograde memory and learning abilities. One year later, the autobiographic memory deficit was unmodified, except for what had been re-learnt. Brain MRI was normal while H20 brain PET scans demonstrated hypometabolism in the right globus pallidus and putamen after 2 weeks from onset, which was no longer present one year later. The absence of a clear pathomechanism underlying focal amnesia lead us to consider this case as an example of functional retrograde amnesia.

## 1. Introduction

Organic amnesia is a syndrome in which the patient shows normal intelligence and cognitive profile associated to different degrees of impairment in anterograde and retrograde memory.

Further relevant information comes from single case studies of patients with memory deficits. In this category we can include *acquired disorders,* brought about by herpes encephalitis, severe hypoxia, certain vascular lesions, deep midline tumors, head injury, basal forebrain lesions or degenerative brain disease. A particular category of organic amnesia is Transient Global Amnesia (TGA) that from a neuropsychological point of view could share some features with other forms of amnesia, but clinically is a well distinct picture.

In some cases amnesia occurs in association with a psychological aetiology: this non-neurological syndrome is variously named as "hysterical amnesia", "psychogenic amnesia", "dissociative amnesia" and, more recently, "functional amnesia" [16,17,22].

We describe a 47-year-old man, who referred to the Emergency Department for sudden global amnesia and left mild motor impairment in the setting of increased arterial blood pressure. Complete neuropsychological tests one week after the acute onset and 2 months later demonstrated a clear retrograde memory deficit contrasting with the preservation of anterograde memory and of learning abilities, which was unchanged one year later, except for what had been re-learnt.

ISSN 0953-4180/07/$17.00 © 2007 – IOS Press and the authors. All rights reserved

## 2. Case history

### *2.1. Initial assessment.*

A.M. is a right handed, 47-yars-old male who works as business consultant (17 years of educational training) and lives with his family (wife and one daughter) in the south of Italy. His developmental history is unremarkable. Family history is positive for high arterial blood pressure and diabetes. Past medical history includes increased arterial blood pressure six years prior admission to our Clinic but this has not been treated and the patient remained asymptomatic. There is no past history of psychiatric abnormalities, significant psycho-pathological traits or alcohol abuse.

The day of referral to the Emergency Department, he woke up as usual, had his breakfast and took his daughter to school. The last call on his mobile phone is reported at 9.30 a.m. From 9.38 a.m. his phone became unreachable. Instead of meeting with a client as scheduled, he travelled for over 1000 Km to Milan, where he arrived after 12 hours. At that point he asked for help to the police, saying that he was confused and he didn't remember where he lived.

He was admitted to the Emergency Department at our Hospital site. When his wife, advised by police about the finding of her husband, reached him at the hospital, he didn't recognize her. Neurological examination at that time revealed mild motor impairment on the left arm. He was restless and confused. Global amnesia for autobiographic memories became evident. He was unable to give his name, address and any information concerning his family and professional activity. He failed to recognize any of his relatives when they arrived and he hadn't a sense of familiarity for them. His blood pressure on admission was 170/120 and failed to decrease despite treatment for 2 hours. Brain CT scan was normal just like echodoppler of carotid and vertebral arteries. EEG recorded bilateral and predominantly frontal slow waves. Brain MRI was normal. Lumbar puncture was normal. The patient underwent a complete neuropsychological evaluation.

### *2.2. Neuropsychological evaluation: general*

Neuropsychological testing was administered by an experienced examiner in a quiet environment in the hospital. Approximately 60 to 90 minutes were needed to administer the tests. It included a screening test for dementia (Mini-Mental State Examination, MMSE [9]), tests of nonverbal reasoning (Raven's Coloured Progressive Matrices, CPM), auditory language comprehension (Token Test), verbal fluency with phonemic and semantic cues, verbal and spatial short-term memory (Digit Span forward and Spatial Span), spatial long term memory (Rey Recall), verbal learning (Serial Position Curve) and constructional abilities (Rey's complex copy) [19].

MMSE was normal. The patient showed a good performance on long term verbal and spatial memory, short term verbal memory and language production. A normal score was also obtained at the Serial Position Curve, following the Capitani et al. standardization [5]. Language comprehension and abstract visuospatial reasoning abilities were at a borderline level. Instead, his scores at the tests that assess short term spatial memory and constructional apraxia were under the score of the normal reference population.

Although general evaluation showed a global cognitive functioning preserved, with only isolated deficits with regard to short term spatial memory and constructional abilities, the patient appeared very confused about his personal history: he didn't remember who he was, where he lived and which was his job. For this reason, it has been considered interesting to analyze thoroughly remote memory.

### *2.3. Neuropsychological evaluation: memory*

In order to investigate semantic memory, the WAIS [26] information subtest was also administered: the performance was comparable to the one of the reference population. Of note, in the Famous Face Recognition Test [19] the patient's score was normal: he was able to indicate, among four alternatives, the face corresponding to the famous name pronounced by the examiner. The patient's autobiographical memory was tested on a formal questionnaire, the Autobiographical Memory Inventory [15]. The AMI investigates personal semantic information and autobiographic episodes recalled from three periods of life: childhood, early adult life and recent life. The same questionnaire was administered to his wife, in order to check the veracity of the patient's recollections.

The patient couldn't remember specific information of his personal history, i.e. which was his job and the name of clients and colleagues, suggesting a semantic memory deficit. In particular, he said to have a son, instead of a daughter. He couldn't explain which were the activities of his job and when he was asked about his degree and the topics he had studied and applied every day in his working activities , nothing but a sensation of distress was reported. Furthermore, he showed a corresponding deficit on episodic memory: with respect to the same topics (family, job and degree), he had no available memories of detailed events. The most intriguing aspect of his assessments was the lack of emotional participation on topics regarding his family: there was no apparent pleasure in spending time with his daughter. At this point additional tests were performed.

First, the ability to name visually presented objects and actions was tested.

The stimuli used in the action-object picture naming task were taken from the Center for Research in Language-International Picture Naming Project corpus [CRL-IPNP, 2]. These were 60 actions and 60 objects, with half (30) of the items in each category being "easy" and half "difficult". The patient scored perfectly on object naming (60/60 items correctly named), and very well on verb naming (59/60 items correctly named).

Second, in order to test the ability to learn and retain items not semantically associated, the list of couple of words taken from Baddeley et al. was administered [1, 21]. Our patient required six trials to correctly recall the eight word-pairs, and his performance after the delay was excellent too (8 items recalled), suggesting good learning and retention abilities.

The patient was discharged in good clinical conditions aside from the memory impairment on his personal history, with Aspirin 300mg qd and diuretics to regulate his arterial blood pressure.

After two weeks the patient was recovered again to undergo a follow-up session. In this circumstance H20 brain PET scans demonstrated hypometabolism in the right globus pallidus and putamen. Neuropsychological evaluation after two months was substantially unmodified, with the exception of recovered constructional abilities and short-term spatial memory.

### *2.4. 9 months follow-up assessment*

When he returned home his behaviour was completely unaffective towards his wife and daughter; he demonstrated no interest in his favourite hobbies (i.e. his boat) and refused to turn over the pages of his wedding photo album, as his wife suggested in order to stimulate his recall. One month later, the patient returned to work, even if he reported difficulties in remembering dates of meetings and specific work-related items concerning taxes (eg VAT calculations). Since the episode he needs to mark each meeting on a diary in order to recall it. Neuropsychological tests were repeated.

The AMI interview underlined the same mnestic gaps as observed few months before. The anterograde verbal and non verbal, long and short term, memory was preserved, even if the patient showed a qualitative different behaviour during the assessment: he seemed more demonstrative and tended to fill mnestic gaps with false memories.

In order to check the presence of frontal implications, the Wisconsin Card Sorting Test [18] was administered: the score obtained by the patient was comparable to the one of the normal population.

## 3. Discussion

We describe a patient who suffered for a sudden amnesia of uncertain nature. Even if he appears to be similar to TGA patients, his clinical features do not endorse this aetiology. First, TGA is often associated to evidence of cerebral anomalies (i.e. hypoperfusion in the right temporal and parietal regions, as in Venneri (1998) [25]), absent in A.M.; second, by definition, the amnesic syndrome have to be "transient", namely the patients completely and spontaneously recover after a certain period of time. In our case, instead, the amnesia is unmodified after one year. Third, in TGA cases the amnesia is not accompanied by loss of personal identity, and the entity of autobiographic amnesia is temporally graded, with old memories being less affected than recent memories. In A.M., by opposite, both these two conditions were unsatisfied.

The most intriguing feature of our patient's history is the selective lasting amnesia for autobiographic events. In fact, not all his past was lost, but only some specific topics. On follow-up assessment, he was able to report some events that he had ignored on initial assessment, but he acknowledged to have re-learnt them.

There is a great difference between "remember" and "know": the first implies the ability to re-experience the event, the second is a quit "cold" recall, which could result from learning, and it is usually devoid of any emotional component [3,6,10,23].

Actually our patient was able to 'know', but he still could not remember.

For what concerns the selectivity of amnesia, studies on patients with different profiles of recollection based on memories' emotional value, investigated the effects of unilateral damage to the medial temporal lobe [4]. A difference based on which side is affected has been found: patients with right temporal lobectomy recall less unpleasant memories when compared with those with left temporal lobectomy. The same results have been demonstrated with respect to unpleasant memories with high intensity of emotional activation, demonstrating a role of emotional components in autobiographic memories recollection. Whether the same distinction can be made in amnesic patients without recognizable cerebral lesions, as A.M., is so far unknown.

The normal MRI data led us to consider the amnestic syndrome as a voluntary behaviour of our patient (malingering). However, there wasn't any direct intelligible reward the patient could obtain from simulation: he had no legal incentives, economic difficulties and psychosocial conflicts. By contrast, both his private and working life have been negatively affected by the event.

A psychogenic origin for the disorder was also considered but our patient shares only one point with those necessary for diagnosis of psychogenic amnesia, that is the sudden fugue from his environment. The remaining criteria are however apparently not fulfilled [11,14]: there is no past history of psychiatric disorders, there are no stressful or conflictual situations declared nor an highly emotional event known. Despite these observations, we must take into consideration the possibility that such informations could be not carefully collected or investigated during the interview with relatives, as indicated by Kopelman (2000) [12].

Without excluding the former hypothesis, but presuming the accuracy of the psycho-social investigation, the most fitting diagnosis seems to be the "functional retrograde amnesia".

In the past, functional amnesia indicated the cases of focal retrograde amnesia (FRA), in which psychogenic origin was suspected or verified, so that this label represented the opposite of organic amnesia. In particular, functional amnesia was invoked when there wasn't any detectable brain lesion supporting the clinical memory deficit.

Recently this distinction has been criticised [7,8,20], and many authors refer to the term "functional" to indicate the block of a function. From this point of view the distinction is between lesional and non lesional aetiology of amnesia, with functional amnesia being applicable when there is no evidence of brain injuries, but a block of a function is suspected, triggered both by physical or psychic trauma [24]. Kopelman [12, 13] in fact, proposed a composite model of autobiographic amnesia that takes into account both organic and psychogenic mechanisms: instead considering the aetiologies as definite categories, the author suggested an interpretation beside a continuum, where "organic" and "psychogenic" are the two edges, and psychosocial factors interact deeply with brain systems.

This case report is specially important for our knowledge on memory organization because of peculiarity of his clinical features: the patient shows a clear and steady autobiographical memory deficit against the absence of cerebral dysfunction in those sites involved in memory processing.

Therefore, this is a clear example that confirm and justify the need of a third nosographic entity between organic and psychogenic amnesia [8]: in this perspective, the term "functional", as formulated before, is a good candidate.

## Acknowledgement

We wish to thank the patient and his wife.
